# Growth hormone therapy and chromosomal mosaicism in turner syndrome: 25 years of growth outcomes in Taiwan

**DOI:** 10.3389/fendo.2025.1640414

**Published:** 2025-10-21

**Authors:** Yu-Ting Chang, Ying-Hua Huang, Fu-Sung Lo

**Affiliations:** ^1^ Division of Pediatric Endocrinology & Genetics, Department of Pediatrics, Chang Gung Memorial Hospital, Chung Gung University College of Medicine, Taoyuan, Taiwan; ^2^ Department of Pediatrics, Taipei Medical University Hospital, Taipei, Taiwan; ^3^ Division of Endocrinology & Genetics, Department of Pediatrics, Kaohsiung Chang Gung Memorial Hospital, Kaohsiung, Taiwan

**Keywords:** turner syndrome, growth hormone therapy, mosaicism, final adult height, estrogen replacement, bone age, growth velocity, SHOX gene

## Abstract

**Background:**

Turner syndrome (TS), a chromosomal disorder affecting females, is commonly associated with short stature due to haploinsufficiency of the SHOX gene. Recombinant human growth hormone (rhGH) and estrogen replacement therapy (ERT) are standard treatments to improve height and induce puberty. However, the impact of chromosomal mosaicism and other clinical variables on long-term growth outcomes remains controversial, particularly in Asian populations.

**Objective:**

To evaluate the influence of karyotype and other clinical predictors on growth velocity and final adult height in Taiwanese patients with TS undergoing rhGH and ERT.

**Methods:**

This 25-year retrospective multicenter study included 107 TS patients treated at three medical centers between 1997 and 2022. Patients were stratified into non-mosaic and mosaic karyotype groups. Growth patterns, treatment duration, and final adult height were assessed. Multivariable linear regression was used to identify predictors of growth outcome, including karyotype, bone age, baseline height, parental heights, IGF-1 levels, and pubertal status.

**Results:**

rhGH therapy began at a mean age of 11.0 ± 2.78 years (non-mosaic: 11.55 ± 2.76; mosaic: 10.50 ± 2.74). Patients with mosaic TS exhibited higher mean growth velocities during rhGH therapy without significant differences (p >0.05). ERT was initiated at a mean age of 15.10 ± 1.76 years in both groups. Heights at ERT initiation were 142.26 cm (non-mosaic) and 144.26 cm (mosaic). However, final adult height did not significantly differ between non-mosaic groups (148.31 ± 5.09 cm) and mosaic (149.39 ± 5.5 cm), respectively (p > 0.05). Regression analysis identified baseline bone age (β = –2.35, p < 0.0001), initial height (β = 0.55, p < 0.0001), and mid-parental height (β = 0.39, p = 0.0056) as significant predictors of final height. Karyotype, IGF-1, and pubertal status were not independently associated with growth outcomes.

**Conclusion:**

Growth hormone treatment in TS is beneficial, but final adult height is complex. Karyotype, including mosaicism, isn’t a primary driver of adult height; instead, factors such as bone age at treatment initiation, pre-treatment height, and mid-parental height are stronger predictors. This emphasizes the need for early diagnosis, individualized treatment plans focusing on clinical assessments, and appropriately timed hormonal interventions.

## Introduction

Turner syndrome (TS) is a chromosomal disorder that exclusively affects females and is characterized by the complete or partial absence of one X chromosome. Clinically, TS manifests with a broad spectrum of physical and developmental features, including short stature, congenital lymphedema, gonadal dysgenesis, primary ovarian insufficiency, cardiovascular abnormalities, and congenital anomalies of the urinary tract (1).

The prevalence of TS is estimated at approximately 1 in 2,500 live-born females (2). Diagnosis is typically confirmed via karyotyping or fluorescence *in situ* hybridization (FISH) targeting the sex chromosomes. Approximately 50% of individuals present with a 45,X karyotype, while the remainder exhibit various forms of chromosomal mosaicism (e.g., 45,X/46,XX; 45,X/46,i(Xq); 45,X/46,X,+mar), as well as structural abnormalities such as ring chromosomes or partial deletions of the X chromosome. Some cases may also harbor Y chromosomal material, which necessitates additional clinical considerations (3).

Short stature is one of the most consistent features of TS, resulting from haploinsufficiency of the SHOX gene located on the pseudoautosomal regions of the sex chromosomes (4, 5). Affected individuals typically exhibit reduced growth velocity throughout childhood and lack the expected prepubertal growth spurt, resulting in significantly reduced adult height if untreated (6, 7).

Recombinant human growth hormone (rhGH) therapy has proven effective in improving growth velocity and final adult height, particularly when initiated in early childhood (8). As the recent clinical practice guidelines recommend the initiation of estrogen replacement therapy (ERT) between 11 and 12 years of age. Estrogen dosage should be increased slowly to adult replacement dosage over 2-4 years. However, ERT also accelerates epiphyseal maturation and closure, potentially limiting height gains if initiated too early (9). Therefore, in individuals with a later diagnosis (>12 years) who have short stature and remaining growth potential, precise timing and dosing of rhGH and ERT are crucial to balance pubertal progression with growth optimization (8–11).

Beyond chromosomal status, additional factors such as parental heights, bone age, baseline stature, pubertal status, and biochemical markers like insulin-like growth factor-1 (IGF-1) may influence treatment response. However, few studies have integrated these parameters in multivariable models, especially within Asian populations.

This study aims to assess the influence of karyotypic variation and clinical predictors on growth outcomes in Taiwanese girls with TS receiving rhGH and ERT, and to identify independent predictors of final adult height using multivariable regression analysis.

## Patients and methods

### Patients

A retrospective review was conducted on the medical records of patients diagnosed with Turner syndrome (TS) who received care at Linkou, Taipei, and Keelung branches of Chang Gung Memorial Hospital between 1997 and 2022. A total of 118 cases were initially identified. One patient was excluded for not receiving recombinant human growth hormone (rhGH) therapy, and three were excluded due to incomplete treatment records. An additional five patients were excluded for having a Y chromosome or SRY gene with indeterminate phenotypes. Ultimately, 107 patients were included in the final analysis: 46 with non-mosaic TS and 61 with mosaic TS (see **Supplementary Figure 1**).

### Methods

Baseline evaluations included age at diagnosis, chronological age, bone age (via radiographs), height, weight, mid-parental height, and relevant laboratory data. Mid-parental height was calculated using the formula:


(Father's height+Mother's height±12 cm)÷2(+12 cm for boys;−12 cm for girls)

Height standard deviation scores (SDS) were calculated using national reference standards for Taiwanese children (12).

Laboratory assessments included serum levels of insulin-like growth factor-1 (IGF-1), follicle-stimulating hormone (FSH), triglycerides, and total cholesterol. IGF-1 was measured using a chemiluminescent immunoassay (IMMULITE 2000, Siemens Medical Diagnostics, Germany). Clinical assessments were based on physical examinations and evaluations of TS-associated anomalies.

All patients received rhGH at a standardized dose of 1 IU/kg/week (0.35 mg/kg/week), in accordance with Taiwan’s National Health Insurance Administration (NHI) guidelines, which require:

- Age of rhGH therapy should be ≥ 6 years.
- Height before rhGH therapy should be below the 3rd percentile.
- Growth velocity < 4 cm/year over a period of 6 months or more before rhGH therapy.
- Bone age before rhGH therapy should be < 14 years.
- rhGH dose ≤ 1 IU/kg/week (0.35 mg/kg/week).

Continuation of rhGH treatment was allowed if:

- Bone age remained < 14 years.
- Growth velocity should be increased by ≥ 2 cm/year in the first year more than before rhGH therapy.
- Growth velocity remained ≥ 4 cm/year from the second year of rhGH therapy onward.

The rhGH formulations used included Saizen (Merck Serono, Germany), Norditropin (Novo Nordisk, Denmark), and Genotropin (Pfizer, USA). All patients were treated for at least one year, with most undergoing 2–4 years of therapy. During the course of treatment, the dosage of growth hormone was kept unchanged.

ERT was initiated at a mean age of 15.1 years due to the later diagnosis (>12 years) who have short stature and remaining growth potential. Estrogen replacement therapy was initiated with oral estradiol valerate at a starting dose of 0.5 mg daily. The dose was gradually titrated over a period of approximately two years to reach 2 mg daily, aiming to mimic the physiologic progression of puberty. Upon the occurrence of breakthrough bleeding, cyclic progesterone was added to the regimen to induce regular withdrawal bleeding and provide endometrial protection.

### Karyotype classification

Patients were categorized based on chromosomal composition into:

- Non-mosaic TS: 45,X.
- Mosaic TS: 45,X/46,X,i(Xq); 45,X/46,XX; 45,X/46,X,r(X); 46,X,del(Xp); 45,X/46,X,+mar; 45,X/47,XXX; 45,X/46,X; 45,X/46,X,del(Xq); 45,X/46,XX,+mar; 45,X/46,XX/47,XXX; 46,XX/46,X,del(Xq).

Majority of patients received standardized rhGH and ERT regimens and were monitored annually for developmental progress and treatment response.

### Statistical analysis

Of the 107 patients, 46 were classified as non-mosaic TS and 61 as mosaic TS. The Shapiro-Wilk test was used to assess the normality of continuous variables. Normally distributed continuous variables are presented as means ± standard deviations; non-normally distributed variables are presented as medians and interquartile ranges. Categorical variables are presented as percentages.

Independent t-tests were used to compare normally distributed continuous variables between groups, while the Mann-Whitney U test was used for non-normally distributed continuous variables. Chi-square tests or Fisher’s exact test were used to compare categorical variables, as appropriate. A p-value < 0.05 was considered statistically significant.

Multivariable linear regression was conducted to identify independent predictors of final adult height, using the following covariates: karyotype (mosaic vs. non-mosaic), paternal height, maternal height, IGF-1 level, bone age at GH initiation, height before GH therapy, and baseline pubertal status. Regression coefficients (β), standard errors (SE), and p-values were reported.

All statistical analyses were performed using SAS version 9.4 (SAS Institute Inc., Cary, NC, USA). This ensured the reproducibility and robust modeling of both unadjusted and adjusted predictors of growth outcomes.

## Results

### Karyotype distribution

Of the 107 patients, 46 were classified as having non-mosaic Turner syndrome, encompassing six distinct karyotypes. The most common karyotype was 45,X, present in 46 patients (43% of the total cohort). The mosaic TS group included 61 patients with a wider spectrum of chromosomal configurations. The most prevalent mosaic karyotypes were:

- 45,X/46,X,i(Xq) (n = 24; 22.4%).
- 45,X/46,XX (n = 12).
- 45,X/46,X,r(X) (n = 6).
- 46,X,del(Xp) (n = 6).
- 45,X/46,X,+mar (n = 5).
- 45,X/47,XXX (n = 3).

A summary of the karyotypic distribution is presented in **Supplementary Figure 2**.

### Baseline characteristics

**Table 1** presents the baseline characteristics. The overall mean age at diagnosis was 9.47 years, with no significant difference between the non-mosaic (10.17 ± 4.95 years) and mosaic (8.95 ± 4.22 years) groups (p > 0.05).

**Table 1:** Comparison of baseline clinical and biochemical characteristics between non-mosaic (n = 46) and mosaic TS patients (n=61) in this study.

Variable	Total (n=107)	Non-mosaic TS (n=46)	Mosaic TS (n=61)	p-value
Age of diagnosis (year)	9.47 ± 4.56	10.17 ± 4.95	8.95 ± 4.22	0.111
Height before rhGHT (cm)	123.30 ± 12.24	124.44 ± 11.51	122.30 ± 12.88	0.380
Height (SDS) before rhGHT	-3.14 ± 0.86	-3.38 ± 0.84	-2.94 ± 0.82	0.051
Weight before rhGHT (kg)	31.03 ± 10.94	31.95 ± 11.33	30.21 ± 10.64	0.514
BMI before rhGHT	19.671 ± 3.9461	19.93 ± 4.137	19.44 ± 3.7985	0.656
Mid-parental height (cm)	157.03 ± 4.52	156.16 ± 4.82	157.65 ± 4.23	0.104
Birth weight (gm)	2853.95 ± 437.58	2922.11 ± 477.83	2803.88 ± 403.18	0.220
Spontaneous pubertal development (Y/N)	29/65	8/32	21/33	0.050
Spontaneous menarche (Y/N)	9/97	2/22	7/54	0.342
Baseline IGF-1 (ng/mL)	233.93 ± 142.76	231.98 ± 163.82	235.34 ± 127.26	0.470
FSH (3.5-12.5mIU/mL)	56.34 ± 47.42	66.54 ± 43.32	46.87 ± 49.57	0.038
Hashimoto thyroiditis (Y/N)	11/83	8/34	3/49	0.058
Heart anomaly (Y/N)	10/81	5/35	5/46	0.744
Kidney anomaly (Y/N)	26/64	14/28	12/36	0.384

rhGHT, recombinant human growth hormone therapy; Y/N, Yes/No.

This table presents baseline data for 107 TS patients categorized into non-mosaic (n = 46) and mosaic (n = 61) groups. Variables include age at diagnosis, anthropometric measures, hormone and lipid profiles, and associated clinical conditions. No statistically significant differences were observed between the two groups across most parameters. A higher prevalence of Hashimoto thyroiditis and kidney anomalies was noted in the non-mosaic group, though these did not reach statistical significance.

Baseline serum IGF-1 levels were similar between groups (non-mosaic: 231.98 ± 163.82 ng/mL; mosaic: 235.34 ± 127.26 ng/mL). FSH levels were elevated in both groups, but significantly higher in the non-mosaic group (66.54 ± 43.32 mIU/mL) compared to the mosaic group (46.87 ± 49.57 mIU/mL, p = 0.0382). Lipid profiles were comparable between groups.

The non-mosaic group had a slightly higher birth weight (2922.11 ± 477.83 g) compared to the mosaic group (2803.88 ± 403.18 g), but this difference was not statistically significant. Mid-parental height and pre-treatment weight were similar across groups. The overall mean baseline height was 123.30 ± 12.24 cm (SDS: -3.14 ± 0.86), with no significant difference between non-mosaic (124.44 ± 11.51 cm, SDS: -3.38 ± 0.84) and mosaic patients (122.30 ± 12.88 cm, SDS: -2.94 ± 0.82).

### Pubertal and clinical features

Before ERT, 20% (8/40) of non-mosaic and 38.9% (21/54) of mosaic patients had spontaneous pubertal development. Spontaneous menarche occurred in 8.3% (2/24) of non-mosaic and 11.4% (7/61) of mosaic patients. Hashimoto thyroiditis was diagnosed in 11 patients (non-mosaic: 8; mosaic: 3). Cardiac anomalies were reported in 11 patients, evenly split between groups. Renal anomalies were more common in the non-mosaic group (33.3%, 14/42) than in the mosaic group (25.0%, 12/48).

The non-mosaic group had a significantly higher percentage of individuals with multiple nevi (63.04%) compared to the mosaic group (24.59%) (x² = 10.21, p = 0.0014). There was no significant difference between the groups in the distribution of puberty stages (x² = 3.84, p = 0.05) or the occurrence of menarche (p = 0.34). There was no significant difference in Hashimoto’s thyroiditis (p = 0.058). Similarly, there were no significant differences in heart conditions (x² = 0.17, p = 0.7439), kidney issues (x² = 0.76, p = 0.3842), micrognathia (x² = 0.39, p = 0.531), a low posterior hairline (x² = 0.64, p = 0.4243), or hyperconvexed nails (x² = 1.17, p = 0.2795). Overall, the two groups were comparable across these characteristics.

### Effect of growth hormone therapy

Growth outcomes are summarized in **Table 2**. GH therapy began at a mean age of 11.0 ± 2.78 years (non-mosaic: 11.22 ± 2.75; mosaic: 10.70 ± 2.83).

**Table 2:** Yearly growth parameters and developmental milestones among patients with non-mosaic and mosaic Turner syndrome receiving recombinant growth hormone (rhGH) therapy.

Parameter	All	All	Non-Mosaic		Mosaic		p-value
	Mean	SD	Mean	SD	Mean	SD	
Age Starting rhGHT (year)	11.0	2.78	11.55	2.76	10.5	2.74	0.067
Height Starting rhGHT	123.3	12.238	124.44	11.51	122.3	12.883	0.380
Weight Starting rhGHT	31.028	10.942	31.949	11.33	30.207	10.64	0.514
BMI Starting rhGHT	19.671	3.9461	19.93	4.137	19.44	3.7985	0.656
BA Starting rhGHT	9.58	2.64	9.89	2.39	9.35	2.83	0.392
After 1 year of rhGHT							
Height (1st Year) (cm)	130.37	11.47	130.83	10.77	129.94	12.18	0.711
Weight (1st Year) (kg)	34.19	10.82	34.54	10.66	33.87	11.09	0.796
BMI (1st Year)	19.606	3.7192	19.684	3.643	19.535	3.8278	0.704
BA (1st Year) (year)	10.16	2.55	10.31	2.51	10.04	2.63	0.690
GV (1st Year) (cm/year)	7.04	1.8	6.78	1.33	7.27	2.13	0.091
After 2 years of rhGHT							
Height (2nd Year) (cm)	134.9	10.75	135.24	10.21	134.59	11.34	0.835
Weight (2nd Year) (kg)	36.76	10.63	37.45	10.45	36.13	10.88	0.493
BMI (2nd Year)	19.801	3.5323	20.062	3.293	19.559	3.766	0.349
GV (2nd Year) (cm/year)	5.64	1.47	5.30	1.45	5.95	1.43	0.055
After 3 years of rhGHT							
Height (3rd Year) (cm)	138.48	10.07	138.74	9.34	138.27	10.75	0.988
Weight (3rd Year) (kg)	39.91	10.74	40.05	10.05	39.81	11.40	0.939
BMI (3rd Year)	20.463	3.4811	20.495	3.255	20.438	3.6995	0.790
GV (3rd Year) (cm/year)	4.72	1.5	4.66	1.25	4.76	1.70	0.796
After 4 years of rhGHT							
Height (4th Year) (cm)	140.63	8.66	140.98	9.15	140.28	8.34	0.792
Weight (4th Year) (kg)	40.48	9.66	41.01	9.91	39.95	9.61	0.560
BMI (4th Year)	20.236	3.2441	20.346	3.04	20.126	3.505	0.568
GV (4th Year) (cm/year)	4.57	1.61	4.15	1.21	4.97	1.85	0.076
Age Starting Estrogen (year)	15.1	1.76	15.04	7.42	15.01	1.69	0.657
Height Starting Estrogen (cm)	143.32	6.25	142.26	6.65	144.26	5.73	0.255
Final adult heigh (cm)	148.83	5.29	148.31	5.09	149.39	5.50	0.387

rhGHT, recombinant human growth hormone therapy, GV, growth velocity; BA, bone age.

This table summarizes height, weight, BMI, growth velocity (GV), and bone age (BA) from the initiation of rhGH therapy through the fourth year, as well as age and height at the start of estrogen therapy. Data are presented as mean ± standard deviation. rhGHT, recombinant human growth hormone therapy; GV, growth velocity; BA, bone age.

• After Year 1:

Height: 130.37 cm; Weight: ~34.19 kg.

Growth velocity: 6.78 cm/year (non-mosaic) vs. 7.27 cm/year (mosaic), p = 0.909.

• After Year 2:

Height: 134.9 cm; Growth velocity: 5.30 cm/year (non-mosaic) vs. 5.95 cm/year (mosaic), p = 0.0547.

• After Year 3:

Height: 138.48 cm; Growth velocity: 4.66 cm/year (non-mosaic) vs. 4.76 cm/year (mosaic), p = 0.7963.

• After Year 4:

Height: 140.63 cm; Growth velocity: 4.15 cm/year (non-mosaic) vs. 4.97 cm/year (mosaic), p = 0.0755.

ERT was initiated at a mean age of 15.10 ± 1.76 years in both groups. Heights at ERT initiation were 142.46 cm (non-mosaic) and 144.26 cm (mosaic). Final adult heights were 148.31 ± 5.09 cm and 149.39 ± 5.50 cm, respectively (p = 0.3874).

### Growth velocity trends

**Figure 1:**
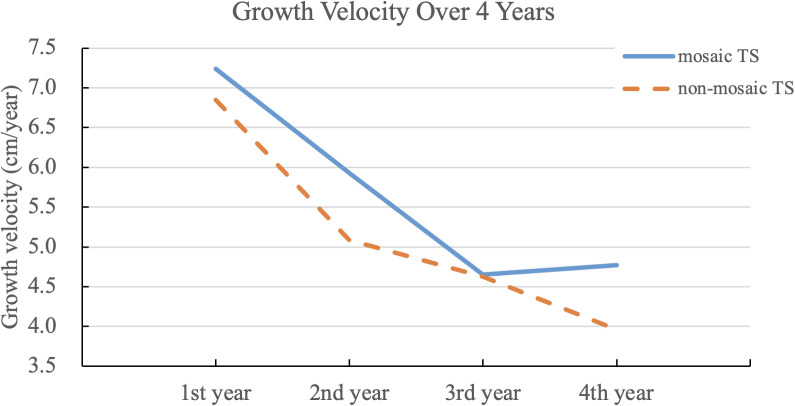
Growth velocity over four years of rhGH therapy in non-mosaic and mosaic Turner syndrome, including data from some individuals who started using ERT. The line graph depicts annual growth velocities following initiation of growth hormone therapy in both non-mosaic and mosaic TS groups. While both groups demonstrated declining growth velocity over time, the mosaic group consistently showed slightly higher values, with a statistically significant difference observed in the second year (p = 0.0137).

As shown in **Figure 1**, the highest growth velocity occurred in the first treatment year, followed by a gradual decline and stabilization at ~4–5 cm/year by year three. The mosaic group demonstrated a slightly higher velocity throughout but without significant difference.

### Predictors of growth outcomes

Multivariable linear regression results are detailed in **Table 3**. The most significant predictors of final adult height were:

**Table 3:** Multivariable linear regression analysis identifying predictors of final adult height in patients with TS.

Variable	Beta (β)	Standard error (SE)	p-value
Mosaic TS	-1.59	1.34	0.2455
Midparental Height	0.39	0.13	0.0056
Baseline IGF-1	-0.01	0.00	0.1194
Initial bone age	-2.35	0.42	<0.0001
Initial height	0.55	0.10	<0.0001
Pubertal Status	-0.04	1.73	0.9838

The table displays regression coefficients (β), standard errors (SE), and p-values for each variable included in the model. Significant predictors of final height included initial bone age (β = –2.375, p < 0.0001), initial height before growth hormone therapy (β = 0.558, p < 0.0001), and midparental height (β = 0.038, p = 0.006). Mosaic karyotype, IGF-1 level, and pubertal status were not significant predictors.

- Initial bone age: β = –2.35, SE = 0.42, p < 0.001.
- Initial height: β = 0.55, SE = 0.10, p < 0.001.
- Midparental height: β = 0.39, SE = 0.13, p = 0.0056.

Baseline IGF-1 level showed trends but was not significant. Neither mosaic karyotype status (β = –1.59, p = 0.2455) nor pubertal status (β = –0.04, p = 0.9836) significantly predicted final height.

## Discussion

Growth failure in Turner syndrome (TS) is primarily due to haploinsufficiency of the short stature homeobox-containing (SHOX) gene located on the short arms of the X and Y chromosomes. Mutations or deletions in SHOX or its regulatory elements result in impaired growth patterns in affected individuals (13–15).

Mosaicism can attenuate these effects. Individuals with mosaic karyotypes (e.g., 45,X/46,XX) may retain partial SHOX gene function, leading to milder short stature and better growth potential compared to those with complete monosomy X (4).

Recombinant human growth hormone (rhGH) therapy has consistently demonstrated efficacy in improving growth velocity and final adult height in girls with TS. Early diagnosis and initiation of therapy are critical for maximizing these outcomes (16, 17). Our findings further affirm that early therapeutic intervention can substantially influence growth trajectories.

Midparental height showed a statistically significant association, highlighting a genetic contribution to growth outcomes. Baseline IGF-1 levels trended toward significance but did not reach statistical thresholds. Mosaic karyotype and pubertal status at baseline did not significantly predict final adult height.

Our findings align with previous reports, including the review by Aversa et al. (1), which concluded that karyotype alone does not predict height prognosis or GH treatment efficacy in TS. These data reinforce the need to prioritize clinical over genetic predictors when developing individualized treatment strategies.

A prior study from Taiwan reported that TS patients treated with a combination of growth hormone and anabolic steroids achieved a height velocity of 7.40 cm/year during the first year and 6.15 cm/year in the second year, resulting in an average final adult height of approximately 150 ± 4.1 cm (18). This historical benchmark helps contextualize our current findings, which were based solely on recombinant human GH therapy without adjunctive anabolic agents.

ERT plays a pivotal role in pubertal development but must be carefully timed to prevent premature epiphyseal closure. Most guidelines suggest delaying ERT until age 13–15 or until bone age reaches 12 years (6, 19–21). Some more recent recommendations advocate for low-dose estrogen initiation between 11–12 years, with gradual dose titration, to balance growth and puberty (21, 22).

This study indicates a later than ideal initiation of both recombinant human growth hormone (rhGH) and estrogen replacement therapy (ERT) in the Taiwanese Turner Syndrome (TS) cohort. The mean age for rhGH therapy initiation was 11.0 years. Under the Taiwanese National Health Insurance regulations, rhGH reimbursement requires fulfillment of strict auxological criteria, including short stature below the 3rd percentile and reduced growth velocity, which may not be evident immediately at diagnosis. Comprehensive baseline assessments may have further extending the timeline.

This study considers mechanisms impacting growth rate and proposes future research. ERT initiation at 15.1 years balances growth potential and pubertal development, influenced by diagnostic and regulatory factors. The proposed mechanism involves mosaic individuals having a subpopulation of cells with two functional SHOX genes, leading to a transient growth advantage. This may trigger earlier epiphyseal maturation, negating long-term benefits. Individual growth potential is strongly influenced by skeletal maturity. Response to ERT may differ in mosaic vs non-mosaic patients. Future studies should include: assessing SHOX expression, advanced imaging of growth plates, pharmacogenomic studies, and controlled ERT studies to better understand final height outcomes.

Several limitations should also be acknowledged. As a retrospective study, the analysis was limited by incomplete documentation in some cases, leading to the exclusion of a small number of patients. The study cohort, while relatively large for a rare condition, remains modest in size compared to international registries, which may limit statistical power for subgroup analyses. In addition, the study lacked a control group of untreated patients, precluding direct estimation of the absolute benefit of rhGH therapy in this population. Information on adherence to therapy, lifestyle factors, and detailed timing or titration of ERT initiation was not consistently available, which may have influenced growth outcomes. Finally, the findings reflect a single ethnic population, and extrapolation to other populations should be made with caution.

This study has several notable strengths. First, it represents the largest retrospective cohort of Taiwanese patients with Turner syndrome treated with recombinant human growth hormone (rhGH) and estrogen replacement therapy (ERT) to date, spanning a 25-year period across three major medical centers. The multicenter design increases the generalizability of the findings within the Taiwanese population. Second, all patients received standardized rhGH dosing according to the National Health Insurance Administration guidelines, which minimizes variability in treatment protocols and enhances internal validity. Third, detailed longitudinal data on growth velocity, bone age, and final adult height allowed for robust evaluation of both short-term treatment responses and long-term outcomes. Finally, the use of multivariable linear regression permitted simultaneous assessment of karyotype and multiple clinical predictors, providing a comprehensive view of the determinants of final height in Turner syndrome.

## Conclusion

This Taiwanese retrospective study and the recent systemic review highlight that growth hormone (GH) treatment in Turner Syndrome (TS) is beneficial, but final adult height is complex and not solely determined by genetics. The studies converge in their finding that karyotype, including mosaicism, isn’t a primary driver of adult height; instead, factors such as bone age at treatment initiation, pre-treatment height, and maternal height are stronger predictors. This emphasizes the need for early diagnosis, individualized treatment plans focusing on clinical assessments, and appropriately timed hormonal interventions, acknowledging that data may be limited in scope, ethnic diversity, and control groups (21). Future prospective studies with larger sample sizes are needed to refine predictive models and further support precision-based approaches in managing TS.

## Data Availability

The original contributions presented in the study are included in the article/**Supplementary Material**. Further inquiries can be directed to the corresponding author.

## Supplementary material

Supplementary Figure 1 Flowchart of patient selection and study cohort formation. A total of 118 patients diagnosed with TS between 1997 and 2022 across three medical centers were retrospectively reviewed. Eleven patients were excluded due to lack of rhGH therapy (n = 1), incomplete treatment documentation (n = 3), presence of Y chromosome or SRY gene with indeterminate phenotype (n = 5), or missing final height data (n = 2). The final study cohort included 107 patients: 46 with non-mosaic TS and 61 with mosaic TS.

Supplementary Figure 2 Distribution of karyotypes among patients with Turner syndrome (TS). This table presents the karyotypic distribution of 107 patients, grouped into non-mosaic and mosaic Turner syndrome classifications. The most frequent non-mosaic karyotype was 45,X (43%), whereas the predominant mosaic form was 45,X/46,X,i(Xq) (22.4%). The frequencies and proportions of each identified karyotype highlight the genetic heterogeneity observed in the study population.

## References

[1] Gravholt CH Viuff MH Brun S Stochholm K Andersen NH. Turner syndrome: mechanisms and management. Nat Rev Endocrinol. (2019) 15:601–14. doi: 10.1038/s41574-019-0224-4, PMID: 31213699

[2] Stochholm K Juul S Juel K Naeraa RW Gravholt CH. Prevalence, incidence, diagnostic delay, and mortality in Turner syndrome. J Clin Endocrinol Metab. (2006) 91:3897–902. doi: 10.1210/jc.2006-0558, PMID: 16849410

[3] Fiot E Alauze B Donadille B Samara-Boustani D Houang M De Filippo G. Turner syndrome: french national diagnosis and care protocol (NDCP; national diagnosis and care protocol). Orphanet J Rare Dis. (2022) 17:261. doi: 10.1186/s13023-022-02423-5, PMID: 35821070 PMC9277788

[4] Sybert VP McCauley E. Turner syndrome. N Engl J Med. (2004) 351:1227–38. doi: 10.1056/NEJMra030360, PMID: 15371580

[5] Lyon AJ Preece MA Grant DB. Growth curve for girls with Turner syndrome. Arch Dis Child. (1985) 60:932–5. doi: 10.1136/adc.60.10.932, PMID: 4062345 PMC1777486

[6] Ranke MB Pflüger H Rosendahl W Stubbe P Enders H Bierich JR. Turner syndrome: spontaneous growth in 150 cases and review of the literature. Eur J Pediatr. (1983) 141:81–8. doi: 10.1007/BF00496795, PMID: 6662146

[7] Massarano AA Brook CG Hindmarsh PC Pringle PJ Teale JD Stanhope R. Growth hormone secretion in Turner’s syndrome and influence of oxandrolone and ethinyl oestradiol. Arch Dis Child. (1989) 64:587–92. doi: 10.1136/adc.64.4.587, PMID: 2751332 PMC1791986

[8] Rosenfeld RG Frane J Attie KM Brasel JA Burstein S Cara JF. Six-year results of a randomized, prospective trial of human growth hormone and oxandrolone in Turner syndrome. J Pediatr. (1992) 121:49–55. doi: 10.1016/S0022-3476(05)82540-5, PMID: 1625092

[9] Saenger P Wikland KA Conway GS Davenport M Gravholt CH Hintz R. Recommendation for the diagnosis and management of Turner. J Clin Endocrinol Metab. (2001) 86:3061–9. doi: 10.1210/jcem.86.7.7683, PMID: 11443168

[10] Ross JL Quigley CA Cao D Feuillan P Kowal K Chipman JJ. Growth hormone plus childhood low-dose estrogen in Turner syndrome. N Engl J Med. (2011) 364:1230–42. doi: 10.1056/NEJMoa1005669, PMID: 21449786 PMC3083123

[11] Gravholt CH Andersen NH Christin-Maitre S Davis SM Duijnhouwer A Gawlik A International Turner Syndrome Consensus Group Backeljauw PF. Clinical practice guidelines for the care of girls and women with Turner syndrome. Eur J Endocrinol. (2024) 190:G53–G151. doi: 10.1093/ejendo/lvae050, PMID: 38748847 PMC11759048

[12] The Department of Physical Education and Sports in the Ministry of Education in Taiwan. 1995 The report of measuring height, weight, and chest circumference among all school students in Taiwan, 28th Ed. Taipei (1995).

[13] Rappold GA Fukami M Niesler B Schiller S Zumkeller W Bettendorf M. Deletions of the homeobox gene SHOX (short stature homeobox) are an important cause of growth failure in children with short stature. J Clin Endocrinol Metab. (2002) 87:1402–6. doi: 10.1210/jcem.87.3.8328, PMID: 11889216

[14] Rappold G Blum WF Shavrikova EP Crowe BJ Roeth R Quigley CA. Genotypes and phenotypes in children with short stature: clinical indicators of SHOX haploinsufficiency. J Med Genet. (2007) 44:306–13. doi: 10.1136/jmg.2006.046581, PMID: 17182655 PMC2597980

[15] Binder G. Short stature due to SHOX deficiency: genotype, phenotype, and therapy. Horm Res Paediatr. (2011) 75:81–9. doi: 10.1159/000324105, PMID: 21325865

[16] Balduccia R Toscano V Larizza D Mangiantini A Galasso C Municchi G. Effects of long-term growth hormone therapy on adrenal steroidogenesis in Turner syndrome. Horm Res. (1997) 49:210–5. doi: 10.1159/000023173, PMID: 9568804

[17] Kristrom B Ankarberg-Lindgren C Barrenäs ML Nilsson KO Albertsson-Wikland K. Normalization of puberty and adult height in girls with Turner syndrome: results of the Swedish Growth Hormone trials initiating transition into adulthood. Front Endocrinol (Lausanne). (2023) 14:1197897. doi: 10.3389/fendo.2023.1197897, PMID: 37529614 PMC10389045

[18] Lee YJ. Growth hormone therapy in Turner syndrome. Acta Paediatr Taiwan. (2000) 41:292–3. doi: 10.7097/APT.200012.0292 11198933

[19] Carel JC Lahlou N Roger M Chaussain JL. Precocious puberty and statural growth. Hum Reprod Update. (2004) 10:135–47. doi: 10.1093/humupd/dmh012, PMID: 15073143

[20] Demetriou E Emans SJ Crigler JF Jr. Final height in estrogen-treated patients with Turner syndrome. Obstet Gynecol. (1984) 64:459–64., PMID: 6091005

[21] Aversa T Li Pomi A Pepe G Corica D Messina MF Coco R. Growth hormone treatment to final height in turner syndrome: systematic review. Clin Ther. (2024) 46:146–53. doi: 10.1016/j.clinthera.2023.12.004, PMID: 38151406

[22] Gawlik A Malecka-Tendera E. Transitions in endocrinology: treatment of Turner’s syndrome during transition. Eur J Endocrinol. (2014) 170:R57–74. doi: 10.1530/EJE-13-0900, PMID: 24225028

