# Supplementary material for: Growth hormone therapy and chromosomal mosaicism in turner syndrome: 25 years of growth outcomes in Taiwan
Source: Front Endocrinol (Lausanne). 2025 Oct 21;16:1640414. doi: 10.3389/fendo.2025.1640414 (PMC12582916; doi:10.3389/fendo.2025.1640414)
Supplement: Supplementary Figure 1 — Flowchart of patient selection and study cohort formation. A total of 118 patients diagnosed with TS between 1997 and 2022 across three medical centers were retrospectively reviewed. Eleven patients were excluded due to lack of rhGH therapy (n = 1), incomplete treatment documentation (n = 3), presence of Y chromosome or SRY gene with indeterminate phenotype (n = 5), or missing final height data (n = 2). The final study cohort included 107 patients: 46 with non-mosaic TS and 61 with mosaic TS. [file DataSheet1.docx]

**Supplement Materials**

**Supplement Figure 1.** Flowchart of patient selection and study cohort formation.
A total of 118 patients diagnosed with TS between 1997 and 2022 across three medical centers were retrospectively reviewed. Eleven patients were excluded due to lack of rhGH therapy (n = 1), incomplete treatment documentation (n = 3), presence of Y chromosome or SRY gene with indeterminate phenotype (n = 5), or missing final height data (n = 2). The final study cohort included 107 patients: 46 with non-mosaic TS and 61 with mosaic TS.


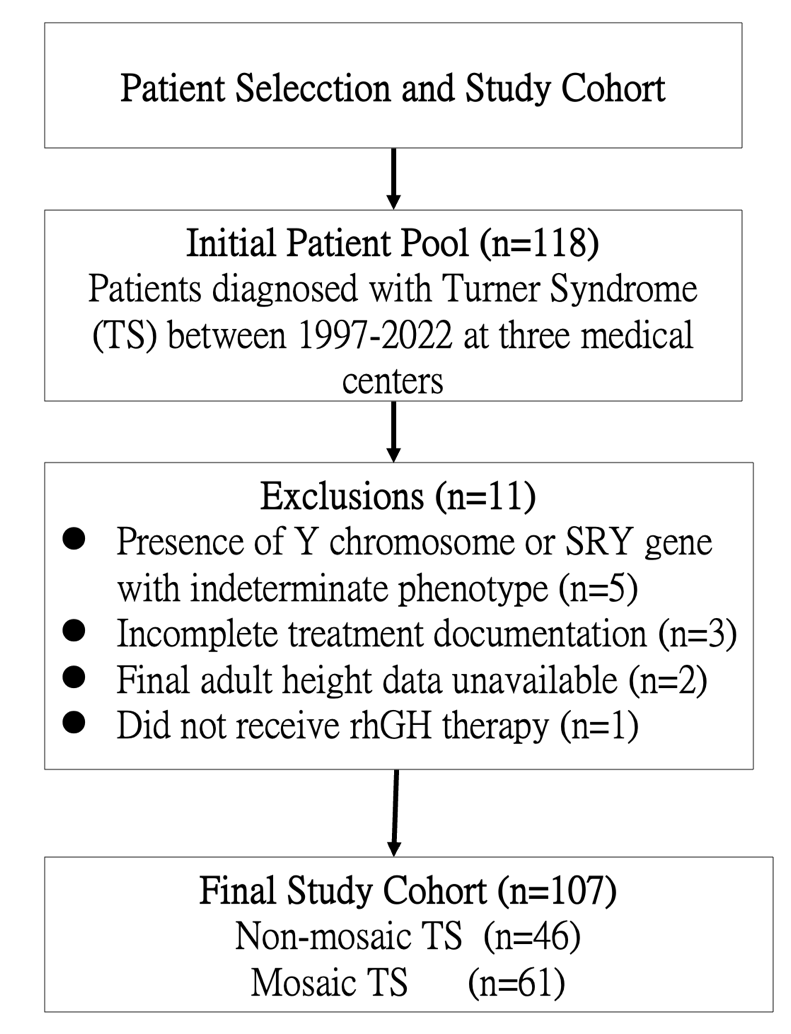


**Supplement Figure 2.** Distribution of karyotypes among patients with Turner syndrome (TS).
This table presents the karyotypic distribution of 107 patients, grouped into non-mosaic and mosaic Turner syndrome classifications. The most frequent non-mosaic karyotype was 45,X (43%), whereas the predominant mosaic form was 45,X/46,X,i(Xq) (22.4%). The frequencies and proportions of each identified karyotype highlight the genetic heterogeneity observed in the study population.

| Karyotype | N | % |
| --- | --- | --- |
| Non-mosaic TS | | |
| 45,X | 46 | 43% |
| Mosaic TS (n=61) | | |
| 45,X/46,X,i(Xq) | 24 | 22.4% |
| 45,X/46,XX | 12 | 11.2% |
| 45,X/46,X,r(X) | 6 | 5.6% |
| 46,X,del(Xp) | 6 | 5.6% |
| 45,X/46,X,+mar | 5 | 4.7% |
| 45,X/47,XXX | 3 | 2.8% |
| 46,X,del(Xp) | 2 | 1.9% |
| 45,X/46,XX,+mar | 1 | 0.9% |
| 45,X/46,XX/47,XXX | 1 | 0.9% |
| 46,XX/46,X,del(Xq) | 1 | 0.9% |
